# Supplementary material for: PD-L1 signaling selectively regulates T cell lymphatic transendothelial migration
Source: Nat Commun. 2022 Apr 21;13:2176. doi: 10.1038/s41467-022-29930-0 (PMC9023578; doi:10.1038/s41467-022-29930-0)
Supplement: Supplementary file 1 — Supplementary Information [file 41467_2022_29930_MOESM1_ESM.pdf]

Supplementary Information

PD-L1 signaling selectively regulates T cell lymphatic transendothelial migration

Piao et al.

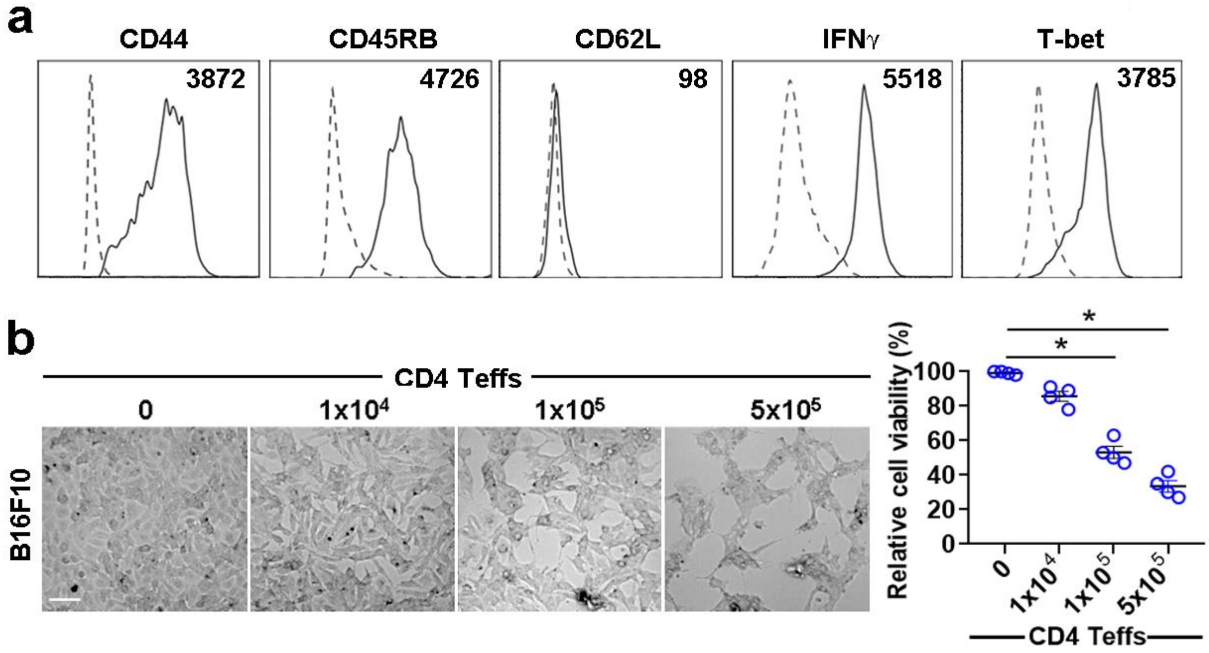

Supplementary Figure 1. Activated Foxp3GFP$^-$CD25$^+$CD4 effector T cells (Teffs) have anti-tumor effector function. a Flow cytometry analysis of CD44, CD45RB, CD62L, IFNγ, and T-bet expression for FACS-sorted Foxp3GFP$^-$CD25$^+$CD4 T cells. **b** Dose-dependent effect of CD4 Teffs on B16F10 melanoma cell viability. Magnification 20 x, scale bar 30 μm. Representative images shown. Data representative of 2 independent experiments. Mean ± SEM. * $p < 0.05$ by one-way ANOVA with Sidak's multiple comparisons test (**b**). Source data are provided as a Source Data file.

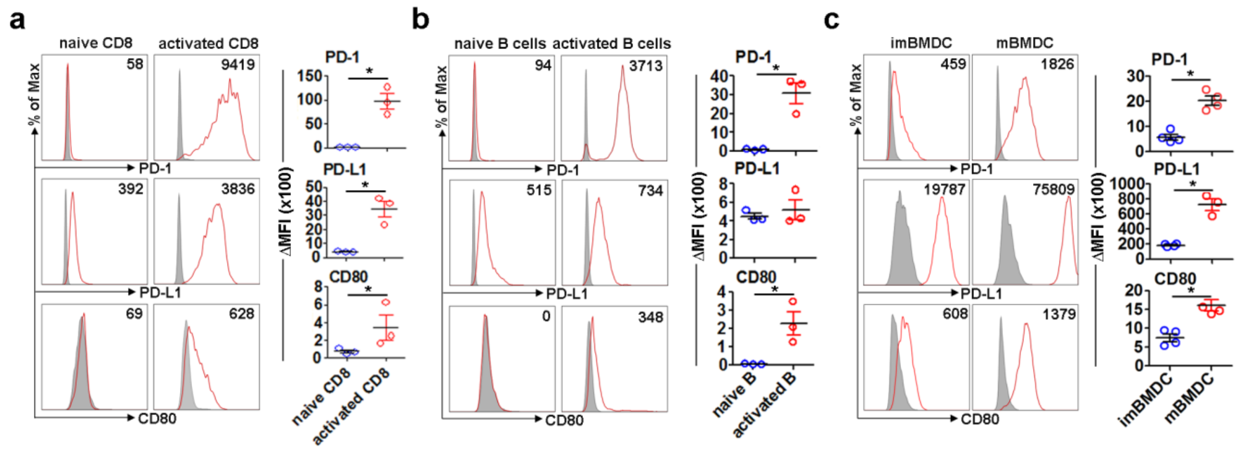

Supplementary Figure 2. Differential expression of PD-1, PD-L1, and CD80 in CD8 T cells, B cells, and BMDCs. a-c Flow cytometry analysis of PD-1, PD-L1, and CD80 expression on mouse naïve and activated CD8 T cells (**a**), B220$^+$ B cells (**b**), and CD11c$^+$ BMDCs (**c**). ΔMFI shown. Data representative of 3 independent experiments. Mean ± SEM. * $p < 0.01$ by unpaired, two-tailed t-test with Welch's correction (**a-c**). Source data are provided as a Source Data file.

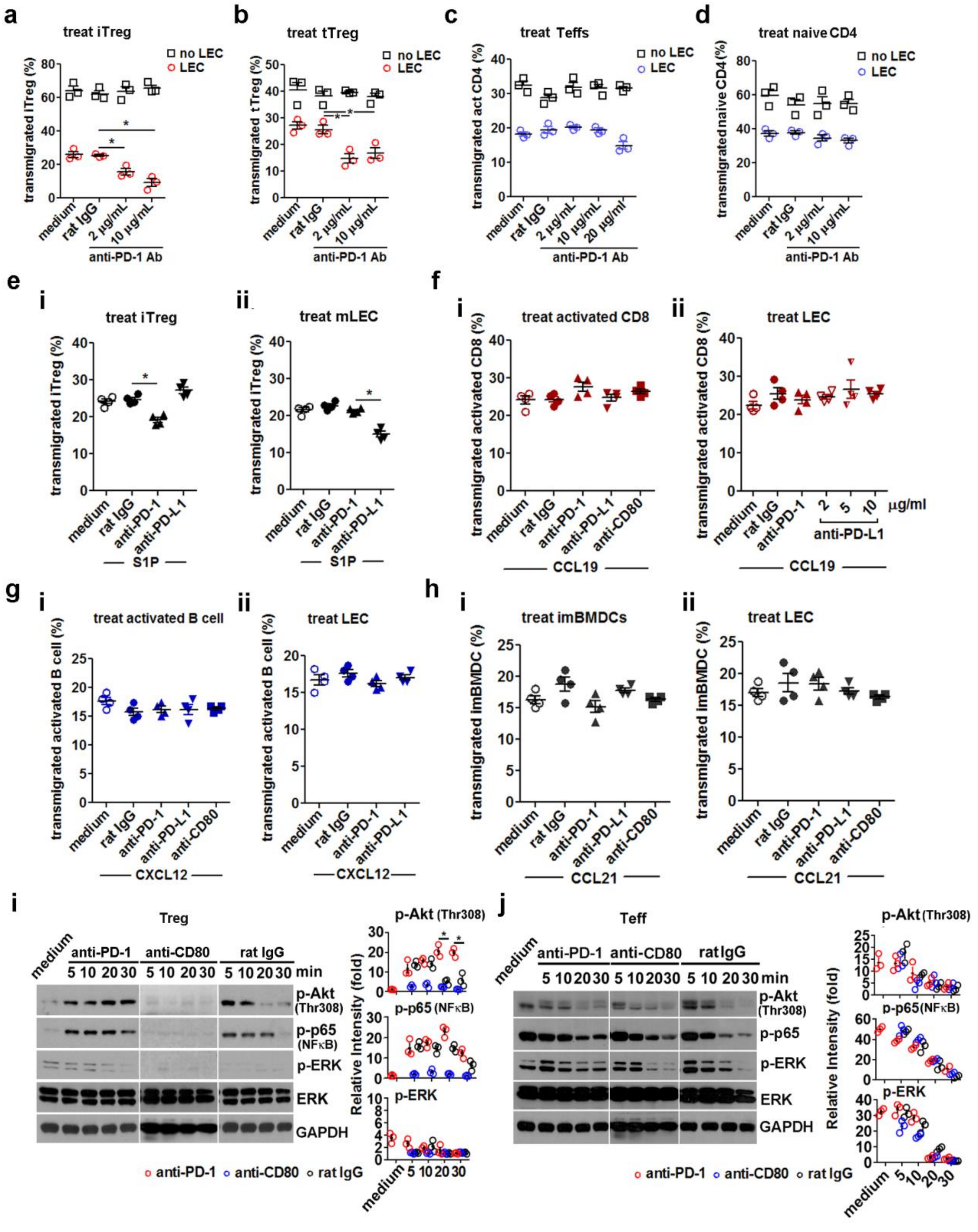

Supplementary Figure 3. Anti-PD-1 mAb inhibits Treg but not CD8 T cell, B cell or BMDC TEM; is dose-dependent; and blocks chemokine- and S1P-driven TEM. a-d TEM of iTregs

(**a**), natural Tregs (**b**), naïve CD4 (**c**), or Teffs (**d**) Cells treated with 2 or 10 μg/ml anti-mouse PD-1 (Rmp1-14), or 2 μg/ml rat IgG (2A3), with or without LEC layers. **e** TEM of iTregs toward S1P. ITregs (**ei**) or LECs (**eii**) treated with anti-mouse PD-1 (Rmp1-14), anti-mouse PD-L1 (10F.9G2), or rat IgG. **f-h** TEM of activated CD8 T cells (**f**), B cells (**g**), and immature BMDCs (**h**). Cells treated as in (**e**) and migrated 3 hours toward mouse CCL19 (**f**), mouse CXCL12 (**g**); 16 hours toward mouse CCL21 (**h**). **i-j** Immunoblots for Akt (Thr308), NFκB-p65, and ERK phosphorylation in iTregs (**i**) or Teffs (**j**) stimulated with anti-mouse PD-1 (Rmp1-14), anti-mouse CD80 (1G10), or rat IgG (2A3) for the indicated times. Relative band intensities shown. Data representative of 3 independent experiments. Mean ± SEM. (**a-j**); * $p < 0.05$ by one-way ANOVA with Sidak's multiple comparisons test (**a-h**) and unpaired, two-tailed t-test with Welch's correction (**i, j**). Source data are provided as a Source Data file.

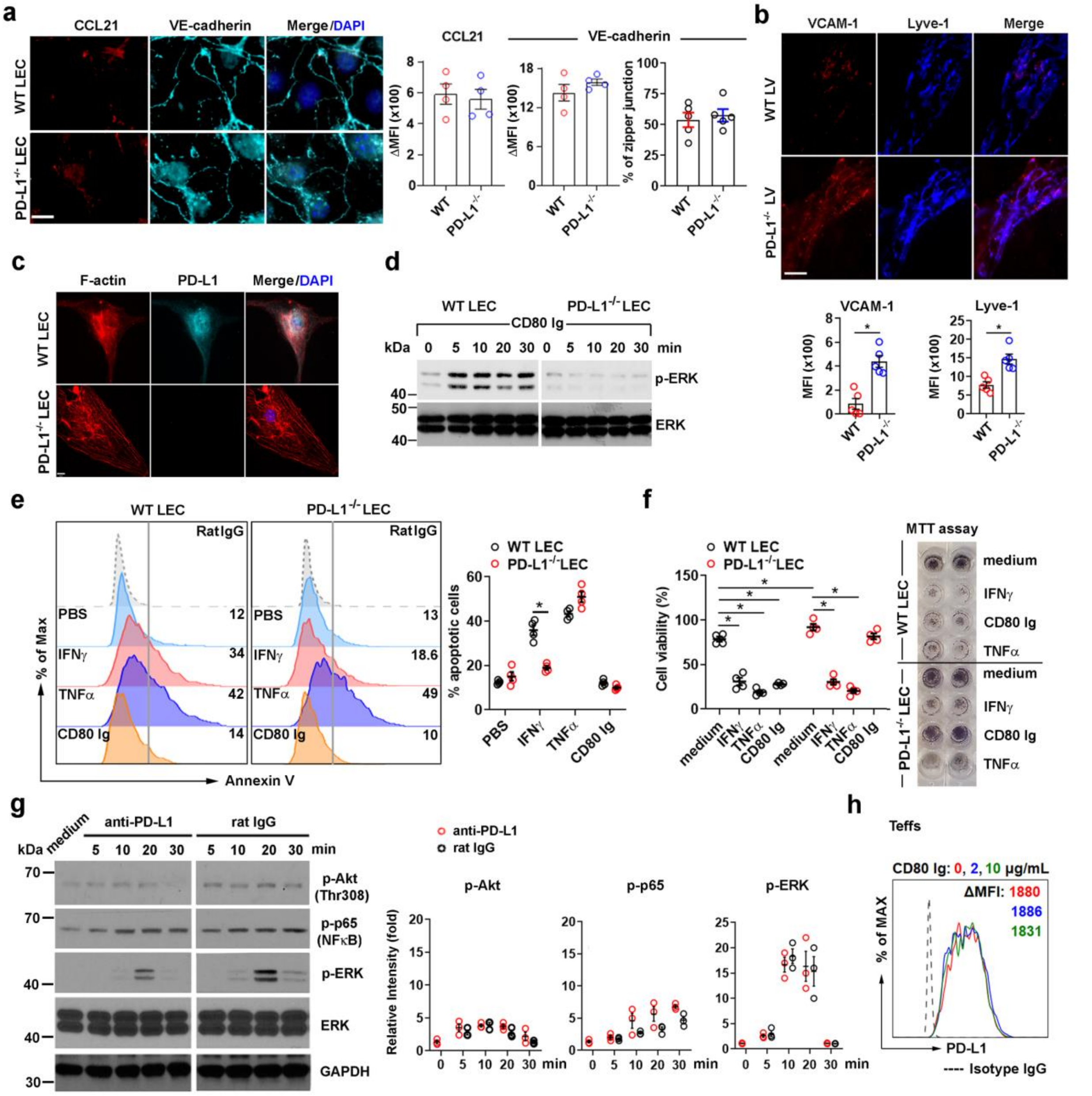

Supplementary Figure 4. PD-L1 signaling regulates lymphatic endothelial cell permeability and viability. a-c Immunohistochemistry for CCL21, VE-cadherin, VCAM-1, Lyve-1, and F-actin in WT or PD-L1$^{-/-}$ LECs in vitro (**a, c**) or lymphatic vessels in vivo (**b**). Magnification 60 x, scale bar 14 μm (a, b) or 8 μm (c). **d** Immunoblots for ERK phosphorylation in WT and PD-L1 $^{-/-}$ LECs. **e-f** Apoptosis and viability of primary LECs treated with 100 ng/mL IFNγ, 20 ng/mL TNFα, or 5

μg/mL CD80 Ig for 36 hours (**e**) or 72 hours (**f**). **g** Immunoblots for Akt (Thr308), NFκB-p65, and ERK phosphorylation in LECs stimulated with 2 μg/mL anti-mouse PD-L1 (10F9G2) or rat isotype IgG2a for the indicated times. Representative blots and relative band intensities (normalized to GAPDH) shown. **h** PD-L1 expression on Teffs treated with increasing dose of CD80 Ig for 1 hour at 37°C. Data representative of 3 (**a-c, g**) or 2 (**d-f, h**) independent experiments. Mean ± SEM (**a, b, e-g**). * $p < 0.01$ versus WT (**a, b, e**) or rat IgG (**g**) by unpaired, two-tailed t-test with Welch's correction, or by one-way ANOVA with Sidak's multiple comparisons test in the same group (**f**). Source data are provided as a Source Data file.

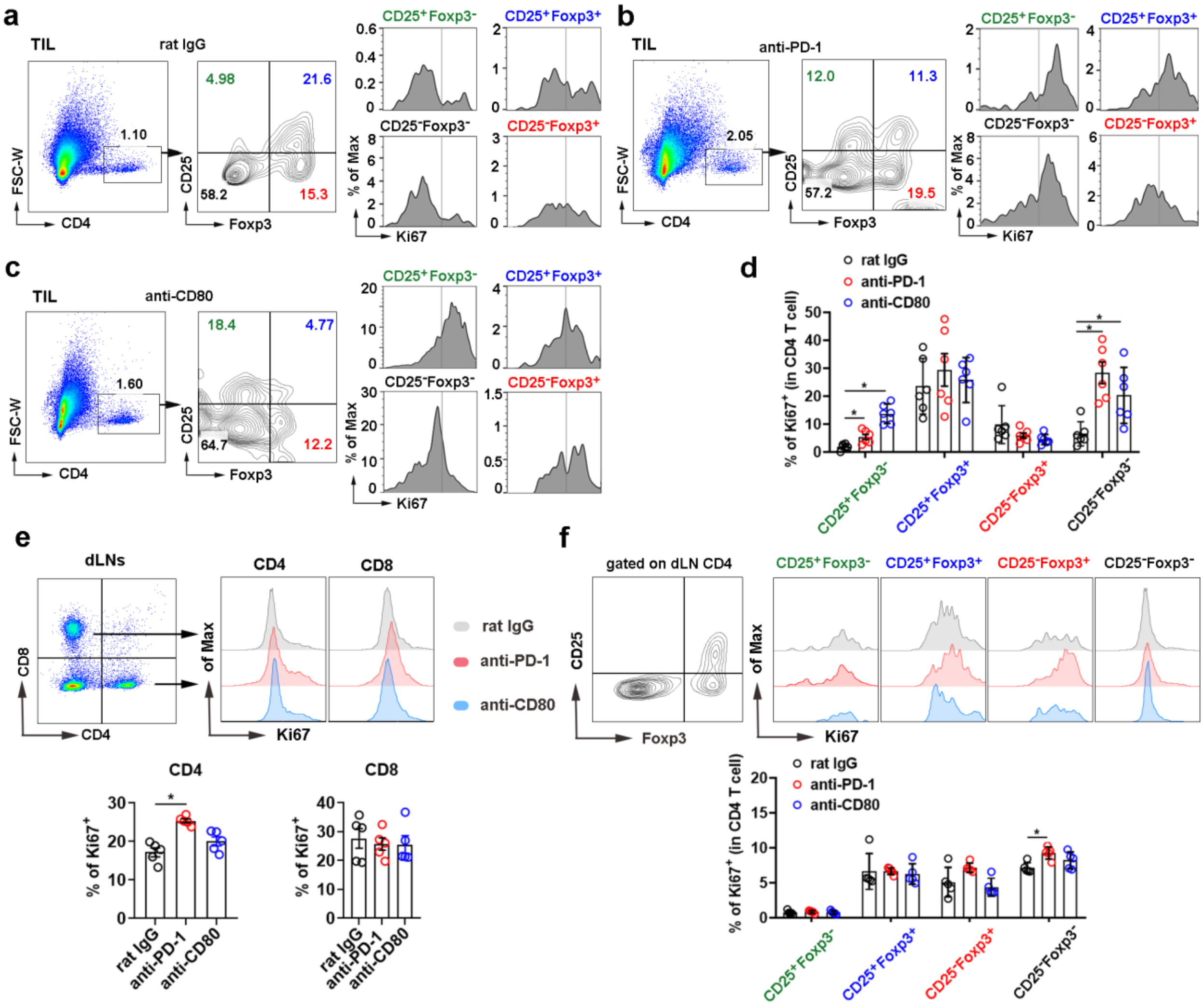

Supplementary Figure 5. Frequencies of Ki67$^+$ T cells in TILs and dLNs of melanoma after PD-1 and CD80 blockade. Flow cytometry analysis of Ki67 expression in CD25$^+$Foxp3$^-$CD4 Teffs, CD25$^+$Foxp3$^+$CD4 Tregs, CD25$^-$Foxp3$^+$Tregs, and CD25$^-$Foxp3$^-$ non-Tregs in TILs (**a-d**) and dLNs (**e-f**) of B16F10 melanoma-bearing mice treated with anti-PD-1 (Rmp1-14), anti-CD80 (1G10) mAbs, or rat IgG isotype. Gating strategy and cell count histograms shown. Ki67 expression in dLN CD4 and CD8 (**e**). Summary of frequencies of CD25$^+$Foxp3$^-$Teffs, CD25$^+$Foxp3$^+$Tregs, CD25$^-$Foxp3$^+$Tregs, and CD25$^-$Foxp3$^-$ non-Tregs in TIL (**d**) and dLN (**f**) CD4 T cells. Data representative of 2 independent experiments. (**d-f**) Mean ± SEM. * $p < 0.05$ by one-

way ANOVA with Sidak's multiple comparisons test. Source data are provided as a Source Data file.

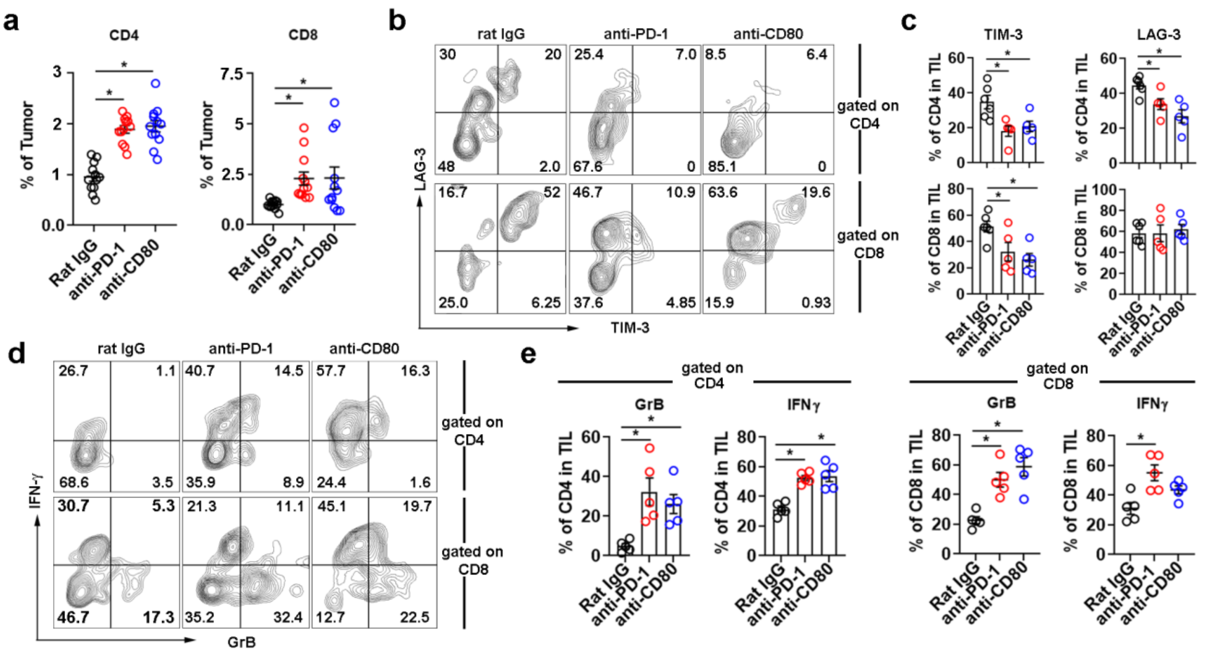

Supplementary Figure 6. PD-1 and CD80 blockade reinvigorate the exhausted T cells. a-e Flow cytometry analysis of CD4 and CD8 in TILs of melanoma-bearing mice treated with anti-PD-1 (Rmp1-14), anti-CD80 (1G10), or rat IgG (2A3). Frequency of CD4 and CD8 in TILs (**a**). LAG-3 and TIM-3 expression in CD4 and CD8 TILs (**b**). Representative dot plots (**b**) and frequencies (**c**). Representative dot plots of intracellular GrB and IFNγ -expressing CD4 or CD8 TILs (**d**). Data representative of 2 independent experiments (5 mice/group). (**a, c, e**) Mean ± SEM. *P < 0.01 by one-way ANOVA with Sidak's multiple comparisons test.

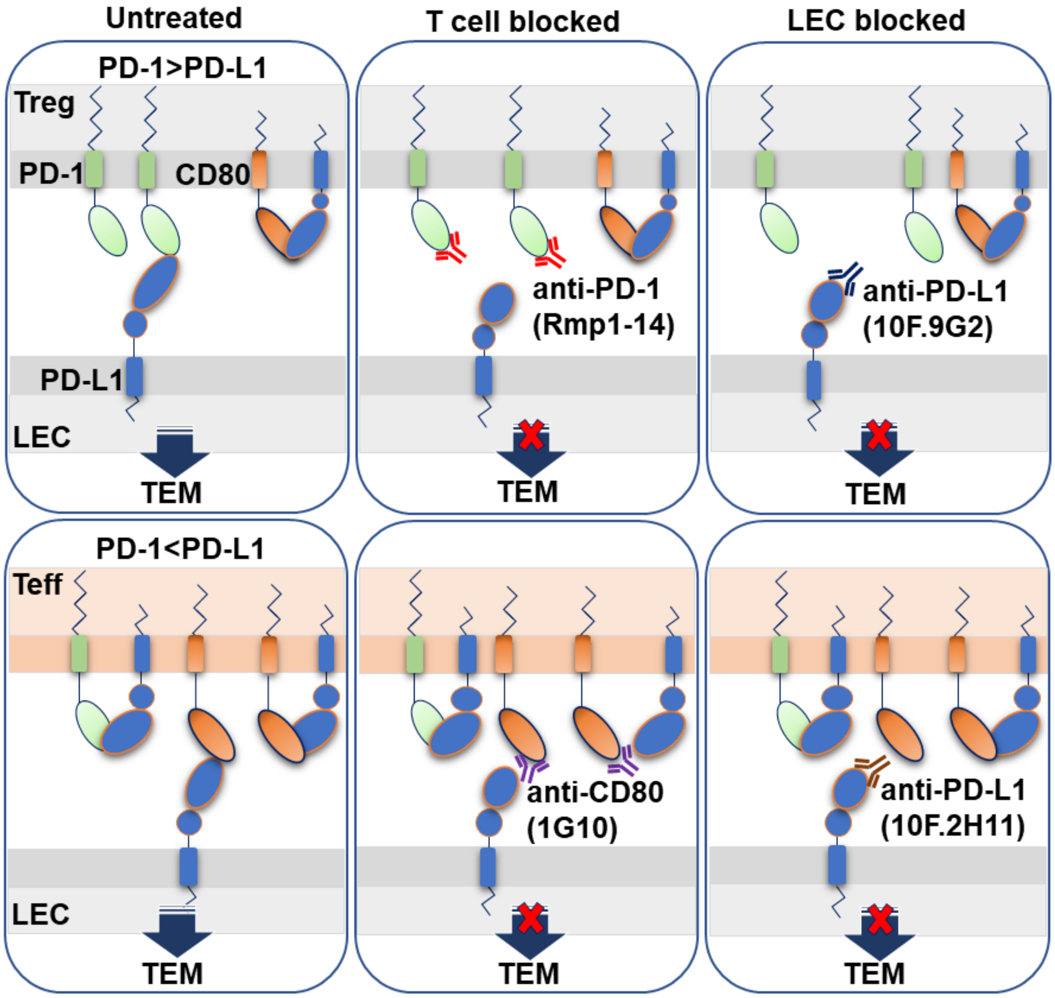

Supplementary Figure 7. Treg PD-1 or Teff CD80 interact with LEC PD-L1 for transendothelial migration (TEM). Activated Treg express higher levels of PD-1 than PD-L1, while Teff express more PD-L1 than PD-1. Conversely, Teff express higher levels of CD80 than Treg. PD-1 or CD80 may bind PD-L1 in cis or trans on T cells, but the function of such T-T interactions is not known. The higher expression of PD-1 enables activated Treg to engage PD-L1 on LEC, and the higher expression of CD80 enables Teff to engage PD-L1 on LEC. PD-1 or CD80 ligation of PD-L1 induces signaling in LEC that leads to structral changes which faciliate TEM. Masking Treg PD-1 or LEC PD-L1 with anti-PD-1 (Rmp1-14) or anti-PD-L1 (10F9G2) Abs, which block the PD-1-PD-L1 interaction, inhibited Treg TEM. Blocking Teff CD80 with anti-

CD80(1G10) or LEC PD-L1 with anti-PD-L1 (10F.2H11), which block the CD80-PD-L1 interaction, inhibited Teff TEM.

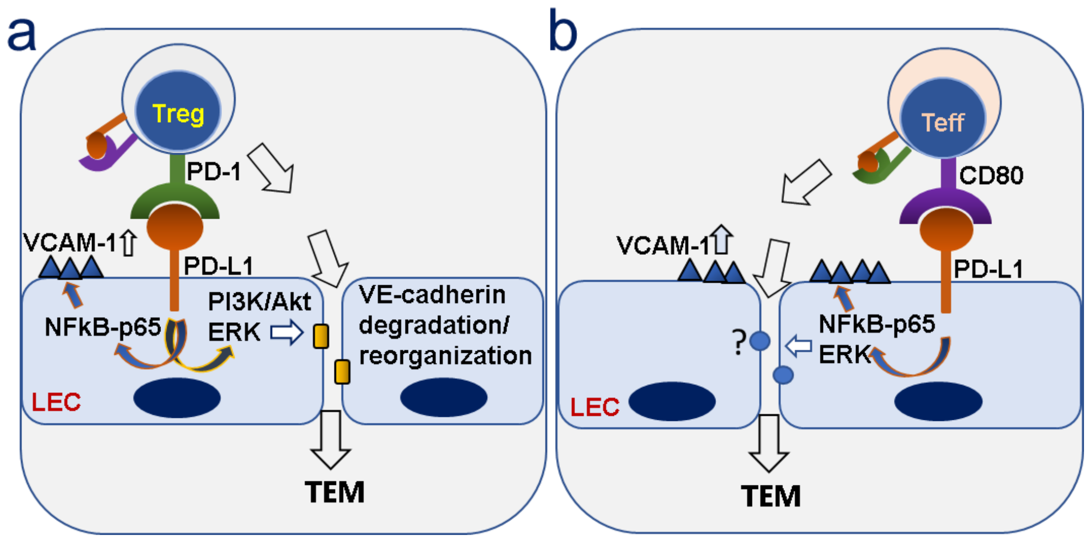

Supplementary Figure 8. Differential PD-L1 signaling by Treg and Teff regulate endothelial structure. **a** PD-1^{high}Treg engage LEC PD-L1 and signal through classical NFκB-p65 to upregulate VCAM-1 and through PI3K/Akt or ERK to downregulate VE-cadherin expression, thus enhancing Treg TEM. **b** PD-1 is occupied in cis by PD-L1 in Teff which expresses higher PD-L1 than PD-1. CD80, with lower PD-L1-binding affinity, is free to engage LEC PD-L1 and signals through NFκB-p65 and ERK pathways to upregulate VCAM-1 expression or other molecules on LEC for increased TEM.

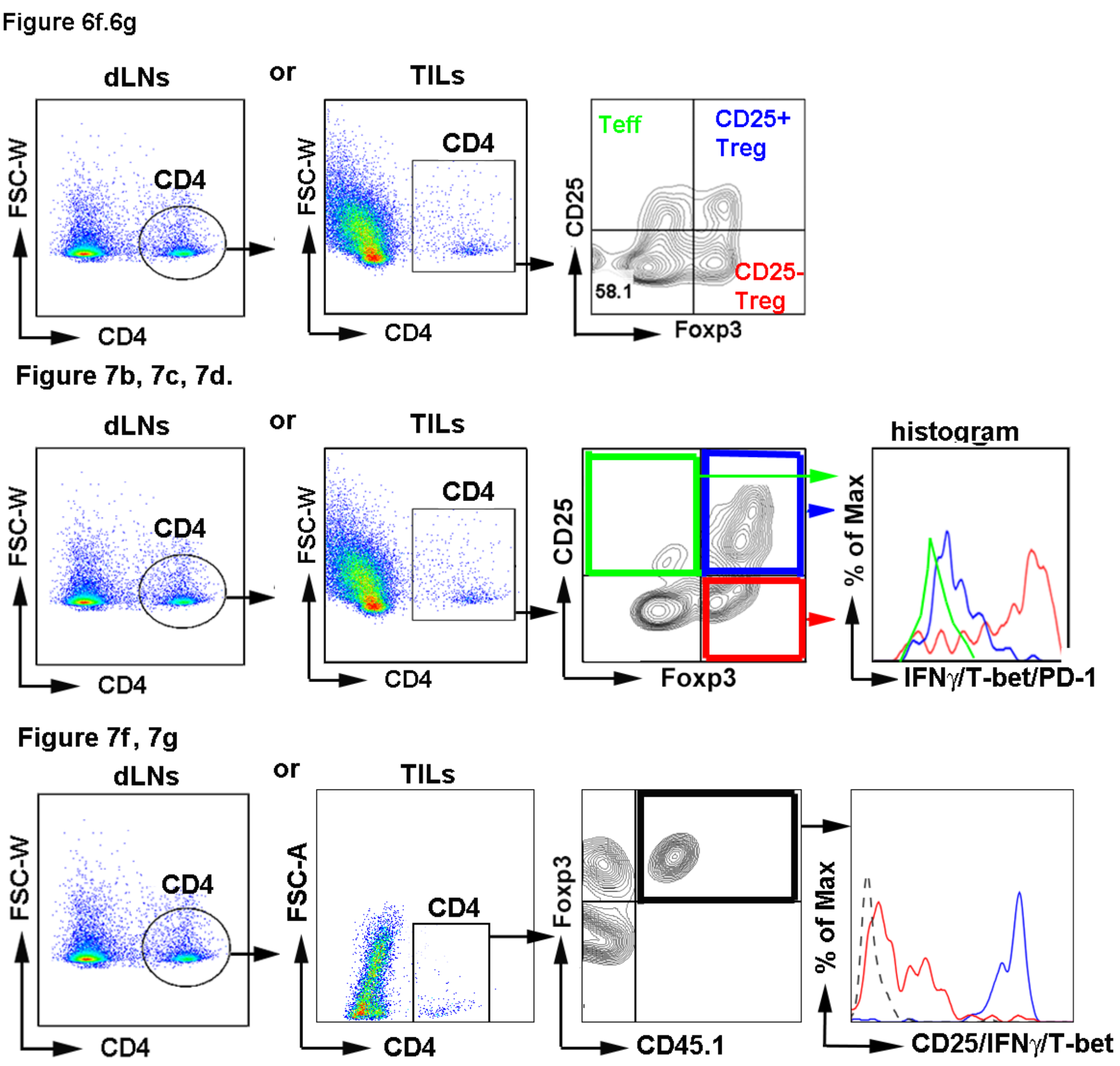

Supplementary figure 9: **Gating strategy for flow cytometry.** Stained single cell suspension from dLNs or tumor TILs was analyzed. Live cells were gated in a Forward scatter (FSC-W or FSC-A) vs. CD4 dot plot. In Figure 6f, g, CD4+ cells were gated for Foxp3 vs CD25 dot plot. In figure 7b-d, each subpopulation: Teff (green), $CD25^{+}$ Treg (blue), and $CD25^{-}$ Treg (red) was further gated for histogram of IFNγ, T-bet, or PD-1 expression. In Figure 7f, g, CD4+ cells were gated for transferred $CD45.1^{+}Foxp3^{+}$Tregs in CD45.1 vs Foxp3 dot plot, The gating were set according to FMO staining.
