# Supplementary material for: PD-L1 signaling selectively regulates T cell lymphatic transendothelial migration
Source: Nat Commun. 2022 Apr 21;13:2176. doi: 10.1038/s41467-022-29930-0 (PMC9023578; doi:10.1038/s41467-022-29930-0)
Supplement: Supplementary file 3 — Reporting summary [file 41467_2022_29930_MOESM3_ESM.pdf]

nature portfolio

Corresponding author(s): Jonathan S. Bromberg

Last updated by author(s): Feb 7, 2022

Reporting Summary

Nature Portfolio wishes to improve the reproducibility of the work that we publish. This form provides structure for consistency and transparency in reporting. For further information on Nature Portfolio policies, see our Editorial Policies and the Editorial Policy Checklist.

Statistics

For all statistical analyses, confirm that the following items are present in the figure legend, table legend, main text, or Methods section.

n/a	Confirmed	
☐	☒	The exact sample size (*n*) for each experimental group/condition, given as a discrete number and unit of measurement
☐	☒	A statement on whether measurements were taken from distinct samples or whether the same sample was measured repeatedly
☐	☒	The statistical test(s) used AND whether they are one- or two-sided<br>*Only common tests should be described solely by name; describe more complex techniques in the Methods section.*
☒	☐	A description of all covariates tested
☐	☒	A description of any assumptions or corrections, such as tests of normality and adjustment for multiple comparisons
☐	☒	A full description of the statistical parameters including central tendency (e.g. means) or other basic estimates (e.g. regression coefficient) AND variation (e.g. standard deviation) or associated estimates of uncertainty (e.g. confidence intervals)
☐	☒	For null hypothesis testing, the test statistic (e.g. *F*, *t*, *r*) with confidence intervals, effect sizes, degrees of freedom and *P* value noted<br>*Give P values as exact values whenever suitable.*
☒	☐	For Bayesian analysis, information on the choice of priors and Markov chain Monte Carlo settings
☒	☐	For hierarchical and complex designs, identification of the appropriate level for tests and full reporting of outcomes
☐	☒	Estimates of effect sizes (e.g. Cohen's *d*, Pearson's *r*), indicating how they were calculated

Our web collection on statistics for biologists contains articles on many of the points above.

Software and code

Policy information about availability of computer code

Data collection	Flow cytometry data were acquired using BD LSR Fortessa (BD Biosciences). Cell migration or tracking were visualized by EVOS FL Auto Cell Imaging System (Thermo Fisher Scientific).
Data analysis	Flow cytometry data were analyzed using FlowJo 10.7.1 (BD). Statistical tests were run using Prism 8.4.1 (GraphPad). Relative band intensities of the blots were measured with ImageJ. Cell tracks were analyzed with Volocity version 6.3 software (Perkin Elmer).

For manuscripts utilizing custom algorithms or software that are central to the research but not yet described in published literature, software must be made available to editors and reviewers. We strongly encourage code deposition in a community repository (e.g. GitHub). See the Nature Portfolio guidelines for submitting code & software for further information.

Data

Policy information about availability of data

All manuscripts must include a data availability statement. This statement should provide the following information, where applicable:

- Accession codes, unique identifiers, or web links for publicly available datasets
- A description of any restrictions on data availability
- For clinical datasets or third party data, please ensure that the statement adheres to our policy

The authors declare that [the/all other] data supporting the findings of this study are available within the paper and its supplementary information files

Field-specific reporting

Please select the one below that is the best fit for your research. If you are not sure, read the appropriate sections before making your selection.

☒ Life sciences ☐ Behavioural & social sciences ☐ Ecological, evolutionary & environmental sciences

For a reference copy of the document with all sections, see nature.com/documents/nr-reporting-summary-flat.pdf

Life sciences study design

All studies must disclose on these points even when the disclosure is negative.

Sample size	sample size were maximized by available resources
Data exclusions	all lymphocytes, macrophages, dendritic cells, or lymphatic endothelial cells were analyzed. No data were excluded from analyses
Replication	Data are reported from at least 3 biological replicates. Replicates were used in experiments as noted in figures. All attempts at replication were successful.
Randomization	Age and sex-matched mice were used for each experiment. Mice studies were randomized.
Blinding	Blinding was no necessary in our study.

Reporting for specific materials, systems and methods

We require information from authors about some types of materials, experimental systems and methods used in many studies. Here, indicate whether each material, system or method listed is relevant to your study. If you are not sure if a list item applies to your research, read the appropriate section before selecting a response.

Materials & experimental systems

n/a	Involved in the study	
☐	☒	Antibodies
☐	☒	Eukaryotic cell lines
☒	☐	Palaeontology and archaeology
☐	☒	Animals and other organisms
☒	☐	Human research participants
☒	☐	Clinical data
☒	☐	Dual use research of concern

Methods

n/a	Involved in the study	
☒	☐	ChIP-seq
☐	☒	Flow cytometry
☒	☐	MRI-based neuroimaging

Antibodies

Antibodies used	Blocking antibodies against mouse PD-1 (Rmp1-14, BE0146, use at final 5 to 20 ug/ml), mouse PD-L1 (10F.9G2, BE0101, use at final 5-20ug/ml), mouse CD80 (1G10, BE0134, use at final 5 to 20 ug/ml) were purchased from BioXCell; GoInVivo purified anti-human PD-1 (EH12.2H7, 329946, use at final 5 to 20 ug/ml), human PD-L1 (29E.2A3, 329728, use at final 5 to 20 ug/ml), and human PD-L1 (MIH3, 374503, use at final 5 to 20 ug/ml) were purchase from Biolegend. All antibodies for flow cytometric analysis were purchased from Biolegend (San Diego, CA) except PE anti-mouse PD-1 (J43, 12-9985-82, 1:400, eBioscience): Alexa Fluor® 647 anti-human PD-1 (EH12.2H7, 329910, 1:300); APC anti-mouse PD-L1 (10F.9G2, 124312, 1:400); Brilliant Violet 421™ anti-human PD-L1 (29E.2A3, 329713, 1:300); FITC anti-mouse CD80 (16-10A1, 104706, 1:300); PE anti-human CD80 (2D10, 305208, 1:300); APC anti-mouse VCAM-1 (429, 105718, 1:100); Brilliant Violet 421™ (RM4-5, 100544, 1:300) or PerCP/Cyanine5.5 anti-mouse CD4 (RM4-5, 100540, 1:400); APC anti-mouse CD25 (PC61, 102012, 1:500); PE anti-mouse Foxp3 (MF-14, 126404, 1:200); APC anti-mouse IFN-y (XMG1.2, 505810, 1:200); FITC anti-T-bet (4B10, 644812,1:200); APC anti-mouse Ki-67 (16A8, 652406, 1:400); PerCP/Cyanine5.5 anti-mouse CD8 (53-6.7, 100734, 1:400); APC anti-human/mouse Granzyme B (QA16A02, 372204, 1:200); APC anti-mouse TIM-3 (B8.2C12, 134008, 1:400); PE anti-mouse LAG-3 (C9B7W, 125208, 1:400), except for PE anti-mouse Lyve-1 (ALY7, 12-044-382, 1:200, eBioscience). Antibodies for Immunohistochemistry: Purified Armenian Hamster anti-mouse PD-1 (J43, 551891, 1:200) or CD80 (16-10A1, 553766, 1:200) and rat anti-mouse PD-L1 (10F9G2, 553766, 1:200), mouse VE-cadherin (11D4.1, 550548, 1:100), or mouse VCAM-1 (429, 550547, 1:200) were purchased from BD Biosciences (Franklin Lakes, NJ); ZO-1 mouse mAb (ZO-1-1A12, 33-910-0, 1:100) and Foxp3 rat mAb (FJK-16s, 50-112-8845, 1:100) from Thermo Fisher Scientific; eFluor 450 anti-mouse Lyve-1 (ALY7, 48-044-382, 1:200, eBioscience); anti-CD4 rabbit mAb (ab183685, 1:200, Abcam, Cambridge, MA); Pacific Blue™ anti-mouse CD25 (PC61, 102022, 1:400, Biolegend). NF-kB p65 (C22B4) rabbit mAb (#4764, 1:1000), F-actin-binding Alexa Fluor® 555 Phalloidin (8953S, 1:40) and the antibodies against phosph-ERK1/2 (Thr202/Tyr 204, 4370s, 1:1000), total ERK1/2 (137F5, 1:1000) , phospho-p65 (Ser536) (93H1, 3033, 1:1000), phospho-Akt (Thr308) (4056s, 1:1000), and GAPDH (2118, 1:1000) were obtained from Cell Signaling (San Diego, CA)
Validation	all the antibodies used are validated by the manufacture and have been reported in publications.

1. anti-mouse PD-1 (Rmp1-14) (nVivoMAb anti-mouse PD-1 (CD279)):https://bxcell.com/product/invivomab-anti-m-pd-1
2.anti-mouse PD-L1 (10F.9G2) (inVivoMAb anti-mouse PD-L1 (B7-H1)): https://bxcell.com/product/m-pdl-1/
3.anti-mouse CD80 (1G10) : https://bxcell.com/product/m-b7-1/
4.GoInVivo purified anti-human PD-1 (EH12.2H7): https://www.biolegend.com/en-us/products/goinvivo-purified-anti-human-cd279-pd-1-antibody-12112?GroupID=BLG5466
5.GoInVivo Brilliant Violet 421™ anti-human PD-L1 (29E.2A3)purified anti-human PD-L1 (29E.2A3) : https://www.biolegend.com/en-us/products/goinvivo-purified-anti-human-cd274-b7-h1-pd-l1-antibody-12695?GroupID=BLG5402
6. Ultra-LEAF™ purified anti-human PD-L1(MIH3):https://www.biolegend.com/en-us/products/ultra-leaf-purified-anti-human-cd274-b7-h1-pd-l1-antibody-14875
7. PE anti-mouse PD-1 (J43): https://www.thermofisher.com/antibody/product/CD279-PD-1-Antibody-clone-J43-Monoclonal/12-9985-82
8. Alexa Fluor® 647 anti-human PD-1 (EH12.2H7):https://www.biolegend.com/en-us/products/alexa-fluor-647-anti-human-cd279-pd-1-antibody-4415
9. APC anti-mouse PD-L1 (10F.9G2): https://www.biolegend.com/en-us/products/apc-anti-mouse-cd274-b7-h1-pd-l1-antibody-6655
10. Brilliant Violet 421™ anti-human PD-L1 (29E.2A3): https://www.biolegend.com/en-us/products/brilliant-violet-421-anti-human-cd274-b7-h1-pd-l1-antibody-7261?GroupID=BLG5402
11. FITC anti-mouse CD80 (16-10A1): https://www.biolegend.com/en-us/products/fitc-anti-mouse-cd80-antibody-41
12. PE anti-human CD80 (2D10): https://www.biolegend.com/en-us/products/pe-anti-human-cd80-antibody-554
13. APC anti-mouse CD106 (429): https://www.biolegend.com/en-us/products/apc-anti-mouse-cd106-antibody-6079
14. PerCP/Cyanine5.5 anti-mouse CD4 (RM4-5): https://www.biolegend.com/en-us/products/percp-cyanine5-5-anti-mouse-cd4-antibody-4230
15. Brilliant Violet 421™anti-mouse CD4 (RM4-5): https://www.biolegend.com/en-us/products/brilliant-violet-421-anti-mouse-cd4-antibody-7349
16. APC anti-mouse CD25 (PC61): https://www.biolegend.com/en-us/products/apc-anti-mouse-cd25-antibody-420
17. PE anti-mouse Foxp3 (MF-14): https://www.biolegend.com/en-us/products/pe-anti-mouse-foxp3-antibody-4660
18. APC anti-mouse IFNg(XMG1.2):https://www.biolegend.com/en-us/products/apc-anti-mouse-ifn-gamma-antibody-993
19: FITC anti-T-bet (4B10): https://www.biolegend.com/en-gb/products/fitc-anti-t-bet-antibody-6435?GroupID=BLG6433
20: APC anti-mouse Ki-67 (16A8): https://www.biolegend.com/en-gb/products/apc-anti-mouse-ki-67-antibody-8447
21. PerCP/Cyanine5.5 anti-mouse CD8 (53-6.7): https://www.biolegend.com/en-gb/products/percp-cyanine5-5-anti-mouse-cd8a-antibody-4255
22. APC anti-human/mouse Granzyme B (QA16A02): https://www.biolegend.com/en-gb/products/apc-anti-human-mouse-granzyme-b-recombinant-antibody-14429
23. APC anti-mouse TIM-3 (B8.2C12): https://www.biolegend.com/en-gb/products/apc-anti-mouse-cd366-tim-3-antibody-9227
24. PE anti-mouse LAG-3 (C9B7W): https://www.biolegend.com/en-gb/products/pe-anti-mouse-cd223-lag-3-antibody-4486
25. PE anti-mouse Lyve-1 (ALY7): https://www.fishersci.com/shop/products/lyve1-rat-anti-mouse-pe-clone-aly7-invitrogen-2/12044382
26.Purified Armenian Hamster anti-mouse PD-1 (J43): https://www.bdbiosciences.com/en-us/search-results?searchKey=Purified%20Armenian%20Hamster%20anti-mouse%20PD-1
27. Purified Armenian Hamster CD80 (16-10A1): https://www.bdbiosciences.com/en-us/search-results?searchKey=Purified%20Armenian%20Hamster%20CD80
28. Purified rat anti-mouse PD-L1 (10F9G2): https://www.bdbiosciences.com/en-us/search-results?searchKey=Purified%20rat%20anti-mouse%20PD-L1
29. Purified mouse VE-cadherin (11D4.1): https://www.bdbiosciences.com/en-us/search-results?searchKey=Purified%20mouse%20VE-cadherin
30.Purified mouse VCAM-1 (429): https://www.bdbiosciences.com/en-us/products/reagents/flow-cytometry-reagents/research-reagents/single-color-antibodies-ruo/purified-rat-anti-mouse-cd106.550547
31. ZO-1 mouse mAb (ZO-1-1A12) : https://www.fishersci.com/shop/products/anti-zo-1-tjp1-clone-zo1-1a12-thermo-scientific-novex/339100#?keyword=anti-mouse%20ZO-1%20mouse%20mAb%20(ZO-1-1A12);
32. Foxp3 rat mAb (FJK-16s) : https://www.fishersci.com/shop/products/foxp3-rat-anti-mouse-rat-clone-fjk-16s-ebioscience/p-7069459#?keyword=Foxp3%20rat%20mAb%20(FJK-16s);
33. eFluor 450 anti-mouse Lyve-1 (ALY7): , eBioscience); https://www.fishersci.com/shop/products/lyve1-rat-anti-mouse-efluor-450-clone-aly7-ebioscience-2/p-7159109#?keyword=eFluor%20450%20anti-mouse%20Lyve-1%20(ALY7);
34. anti-CD4 rabbit mAb (ab183685): https://www.abcam.com/cd4-antibody-epr19514-ab183685.html;
35. Pacific Blue™ anti-mouse CD25 (PC61, Biolegend): https://www.biolegend.com/en-us/products/pacific-blue-anti-mouse-cd25-antibody-3315;
36. NF-kB p65 (C22B4) rabbit mAb (#4764): https://www.cellsignal.com/products/primary-antibodies/nf-kb-p65-c22b4-rabbit-mab/4764
37. F-actin-binding Alexa Fluor® 555 Phalloidin:https://www.cellsignal.com/products/buffers-dyes/alexa-fluor-555-phalloidin/8953?site-search-type=Products&N=4294956287&Ntt=f-actin-binding+alexa+fluor%C2%AE+555+phalloidin&fromPage=plp
38. phosph-ERK1/2 (Thr202/Tyr 204): https://www.cellsignal.com/products/primary-antibodies/phospho-p44-42-mapk-erk1-2-thr202-tyr204-d13-14-4e-xp-rabbit-mab/4370
39. ERK1/2 (137F5): https://www.cellsignal.com/products/primary-antibodies/p44-42-mapk-erk1-2-137f5-rabbit-mab/4695
40. phospho-p65 (Ser536)(93H1): https://www.cellsignal.com/products/primary-antibodies/phospho-nf-kb-p65-ser536-93h1-rabbit-mab/3033
40. phospho-Akt (Thr308) (244F9): https://www.cellsignal.com/products/primary-antibodies/phospho-akt-thr308-244f9-rabbit-mab/4056
41. GAPDH (2118):https://www.cellsignal.com/products/primary-antibodies/gapdh-14c10-rabbit-mab/2118

Eukaryotic cell lines

Policy information about cell lines

Cell line source(s)	Primary dermal LECs of C57BL/6 mouse (cat#C57-6064L, Cell Biologics, Inc) or human (Cat#H-6064L, Cell Biologics, Inc) were from Cell Biologics, Inc. (Chicago, IL); B16F10-Fluc-Neo/eGFP-Puro (cat#CL068, Imanis LIFE SCIENCE)
Authentication	1. C57BL/6 MOUSE PRIMARY DERMAL LYMPHATIC ENDOTHELIAL CELLS: https://cellbiologics.com/index.php?route=product/product&path=2_47_89_91&product_id=2252<br>2. HUMAN PRIMARY DERMAL LYMPHATIC ENDOTHELIAL CELLS: https://cellbiologics.com/index.php?route=product/product&keyword=H-6064L&category_id=0&product_id=2352<br>3. B16F10-Fluc-Neo/eGFP-Puro: https://imanislife.com/products/b16f10-fluc-neoegfp-puro/
Mycoplasma contamination	No mycoplasma contamination was detected.
Commonly misidentified lines (See ICLAC register)	No commonly misidentified lines were used.

Animals and other organisms

Policy information about studies involving animals; ARRIVE guidelines recommended for reporting animal research

Laboratory animals	C57BL/6J (45.1 and 45.2, female, 7–10 weeks old) mice were purchased from The Jackson Laboratory (Bar Harbor, ME). C57BL/6.Foxp3GFP mice were kindly provided by Dr. A. Rudensky (Memorial Sloan Kettering Cancer Center).
Wild animals	no wild animals were used in the study.
Field-collected samples	no field-collected samples were used in the study.
Ethics oversight	All animal experiments were performed in accordance with Institutional Animal Care and Use Committee approved protocols, University of Maryland at Baltimore.

Note that full information on the approval of the study protocol must also be provided in the manuscript.

Flow Cytometry

Plots

Confirm that:

- [x] The axis labels state the marker and fluorochrome used (e.g. CD4-FITC).
- [x] The axis scales are clearly visible. Include numbers along axes only for bottom left plot of group (a 'group' is an analysis of identical markers).
- [x] All plots are contour plots with outliers or pseudocolor plots.
- [x] A numerical value for number of cells or percentage (with statistics) is provided.

Methodology

Sample preparation	Cells were incubated with antibodies for flow cytometry for 30minutes at 4°C, washed with PBS and fixed with 4% paraformaldehyde before analysis. In some cases, cells were fixed and permeabilized and stained with antibodies.
Instrument	Stained cells were analyzed by LSR Fortessa flow cytometer (BD Biosciences). Cells were sorted using a FACS Aria II (BD Biosciences, San Jose, CA)
Software	Results were analyzed with FlowJo 10.7 (Treestar).
Cell population abundance	a minimum of 2 million cell fractions were sorted with >98 % purity and viability.
Gating strategy	live cells were first gated with FSC/SSC, then gating the single population of CD4 T cells, or CD45.1 and CD4 double positive population, followed by histogram of particular subpopulation or intracellular cytokine expressions.

- [x] Tick this box to confirm that a figure exemplifying the gating strategy is provided in the Supplementary Information.
